# Opportunistic illnesses in Brazilian children with AIDS: results from two national cohort studies, 1983-2007

**DOI:** 10.1186/1742-6405-8-23

**Published:** 2011-07-18

**Authors:** Alberto N Ramos, Luiza H Matida, Norman Hearst, Jorg Heukelbach

**Affiliations:** 1 Department of Community Health, School of Medicine, Federal University of Ceará, Rua Professor Costa Mendes 1608, 5° andar-Bloco Didático-Rodolfo Teófilo; CE-CEP: 60430-140, Fortaleza, Ceará, Brazil; 2 STD/AIDS State Program, São Paulo, Brazil; 3 Department of Family and Community Medicine, University of California, San Francisco, USA

## Abstract

**Background:**

HAART has significantly reduced AIDS-related morbidity in children. However, limited evidence is available from developing countries regarding patterns of opportunistic illnesses. We describe these events and their associated factors in children with AIDS in Brazil.

**Methods:**

This study is based on two representative retrospective multi-center cohorts including a total 1,859 children with AIDS, infected via mother-to-child transmission (MTCT), between 1983-2002. Opportunistic illnesses were described and analyzed over time. The association of demographic, clinical and operational data with the occurrence of opportunistic diseases was assessed.

**Results:**

In total, 1,218 (65.5%) had at least one event of an opportunistic disease. Variables significantly associated with occurrence of these events included: region of residence (OR 2.68-11.33, as compared to the Northern region), age < 1 year at diagnosis (OR 2.56, 95% CI 1.81-3.61, p < 0.001), and non-performance of MTCT prevention measures (OR 1.58, 95% CI 1.21-2.07, p < 0.001). Protective factors included year of HIV diagnosis in the HAART era (OR 0.34, 95% CI 0.15-0.76, p = 0.009) and ART use (OR 0.58, 95% CI 0.44-0.77, p < 0.001). In both periods bacterial infections represented the most common opportunistic events (58.6 vs. 34.7%; p < 0.001), followed by *Pneumocystis jirovecii *pneumonia (21.9 vs. 13.2%; p < 0.001), and bacterial meningitis/sepsis (16.8 vs. 7.4%; p < 0.001).

**Conclusions:**

Despite the significant reduction in recent years, opportunistic illnesses are still common in Brazilian children with AIDS in the HAART era, especially bacterial diseases. The data reinforce the need for scaling up prevention of MTCT, early diagnosis of infection, and improvement of comprehensive pediatric care.

## Background

Over the past 15 years, both morbidity and mortality associated with human immunodeficiency virus (HIV) have been systematically reduced in children. Greater knowledge on the clinical course, early diagnosis of HIV infection, adoption of prophylaxis, improved management of opportunistic diseases, and introduction of antiretroviral therapy (ART) contributed to this trend [1-4]. New patterns of morbidity associated with chronic diseases have been described recently in HIV-infected individuals [5-7].

The adoption of highly active antiretroviral therapy (HAART) also has had an important role in modifying the natural history of HIV infection in children, characterized by a reduction of the progression to AIDS. The limited evidence available in children exposed to HIV via mother to child transmission (MTCT) shows reduced rates of clinical events in category "C" of CDC revised classification system for HIV infection. Early diagnosis of HIV infection and HAART has led to a reduction in the frequency of opportunistic diseases in children with subsequent reduced number of hospitalizations [4,8-10]. However, severe bacterial infections, mostly pneumonia, were still observed, even in children without severe immunosuppression [11].

Brazil stands out among the developing countries due to its sustained policy of HIV control, with a history of 25 years of a national control program [6]. Since 1996, the country guarantees free and universal access to antiretroviral drugs [6,12]. Additionally, there is an established health service network for qualified management of HIV infection and opportunistic infections [13]. Since the beginning of the HAART era, significant impacts were observed in Brazil, including a significant reduction of MTCT, AIDS-related hospital admissions, morbidity and mortality. Since 1996, there was a downward trend in mortality in this population corresponding to a relative reduction of 63.6% in all children and 71.2% in children under 5 years of age [6]. However, few children, especially those from highly vulnerable families still acquire HIV from their mothers and may develop AIDS. Two national cohort studies have shown an increased survival of these children with AIDS [14-17]. The probability of survival 60 months after diagnosis, which was 53% among those diagnosed with AIDS between 1983 and 1998, increased to 88% between 1999 and 2002 [14].

Few studies have evaluated changes of clinical patterns with the introduction of HAART in children [18]. The aim of this study is to determine the global and specific frequencies of AIDS-defining illnesses and associated factors in the two national Brazilian cohorts of children with AIDS.

## Methods

### Study Design and Population

This study is based on analysis of two representative multicenter retrospective cohort studies on survival in Brazilian children with AIDS, infected by mother-to-child transmission (MTCT). Both studies followed similar methodological procedures, as described in detail previously [14-17]. Children with HIV/AIDS were defined as those under 13 years of age, based on the national AIDS case definitions in children [6]. In the first cohort, AIDS was defined by the 1994 Brazilian criteria (modified CDC definition and confirmation by signs) [19]; in the second, by the 2003 Brazilian criteria (modified CDC definition [clinical immunodeficiency], confirmation by signs, CD4 criterion [laboratorial immunodeficiency-lymphocyte count, by age strata], death exceptional criterion and death criterion) [6,20].

The first study included 914 Brazilian children with AIDS diagnosed between January 1, 1983 and December 31, 1998. The status of children (dead or alive) was based on the follow-up until death or the end of 2002 [15,17]. The second study was composed of 945 children with AIDS diagnosed between January 1, 1999 and December 31, 2002, followed up until death or the end of 2007 [14,16].

### Data analysis

We considered the first clinical event (primary episode) of each opportunistic illness associated with AIDS in any time of the children follow-up. Based on the Centers for Disease Control and Prevention (CDC) definition [21] and on the Brazilian guidelines [20], we included 20 severe opportunistic diseases indicative of AIDS (category "C"-CDC revised classification system for HIV infection). The opportunistic diseases considered for analysis are depicted in the Table 1.

**Table 1:** Listing and characteristics of selected AIDS-defining illness in children from the two Brazilian cohorts

AIDS-Defining Illness	Characteristics
AIDS Wasting syndrome	HIV-related
Bacterial infections (excluding meningitis or sepsis)	Severe, multiple or recurrent (at least 2 confirmed episodes, 2-year): pneumonia, bone/joint infection, abscess of internal organs, excluding otitis media, abscesses of skin and mucous membranes and catheter-related infections
Bacterial meningitis or sepsis	Severe, multiple or recurrent (at least 2 confirmed episodes, 2-year)
Candidiasis	Esophageal, trachea, bronchi or lungs
Coccidioidomicosis	Disseminated
Cryptococcosis	Extrapulmonary
Cryptosporidiosis	Chronic intestinal, with diarrhea persisting for > 1 month
Cytomegalovirus disease	Onset of symptoms after 1 month of age, at a site other than liver, spleen or lymph nodes
Disseminated mycobacterial disease	*Mycobacterium avium *complex or disease caused by *Mycobacterium kansasii*, at a site other than lungs, skin or cervical/hilar lymph nodes (except tuberculosis or Hansen's disease)
Encephalopathy	HIV-related
Herpes simplex virus	Chronic ulcer(s) in the bronchi, lungs or gastrointestinal tract
Herpes simplex virus, mucocutaneous	Persisting for > 1 month affecting children > 1 month of age
Disseminated histoplasmosis	At a site other than exclusively pulmonary or cervical/hilar lymph nodes
Isosporiasis	Chronic intestinal, persisting for > 1 month
Kaposi's sarcoma	Human herpes virus 8-related
Lymphoma	Primary, in brain or lymphoma, non-Hodgkin B-cell lymphoma (unknown immunologic phenotype) and other lymphomas: large or small, non-cleaved cells (Burkitt or non-Burkitt) or immunoblastic malignant lymphoma no further specified
*Pneumocystis jirovecii *pneumonia	Interstitial pneumonia
Progressive multifocal leukoencephalopathy	JC virus-related
Salmonella	Sepsis, recurrent (non-typhoid)
Toxoplasmosis of brain	Encephalitis with onset after 1 month of age

Clinical events were diagnosed by pediatricians with experience in management of HIV-1 infection, and data were registered in medical records. We reviewed retrospectively these medical records regarding the clinical events. To establish the diagnosis, we followed the Brazilian criteria for definition of AIDS cases in children, based on presumptive and/or definitive diagnosis [20]. We analyzed separately invasive bacterial infection, including or not bacterial meningitis or sepsis. The first group represents largely cases with severe pneumonia.

We verified the occurrence of these opportunistic diseases in the pre-HAART (< 1995) and HAART eras (1996-2007), using 1996 as a cut point. Odds ratios (OR) with their respective 95% confidence intervals (95% CI) were calculated for quantitative comparison of relative frequencies. Additionally, we analyzed variables possibly associated with the occurrence of AIDS-defining diseases. Brazilian regions (child' residence), gender, birth cohort (< 1988, 1989-1995, and 1996-2002), age group (< 1, 1-5, and 6-12 years of age in HIV diagnosis), ART use (in any time), year of ART initiation (< 1996, and 1996-2002), vital status (alive or dead), prenatal care, breast-feeding, and MTCT prophylaxis. Chi-squared and Fisher's exact tests were used where applicable. Statistical analysis was done with the STATA 11.0 software package (Stata Corporation, College Station, USA).

### Ethical considerations

Both studies were approved by the Ethical Review Board of the Center for Reference and Training in STD/AIDS of São Paulo State, following the guidelines of the National Health Council.

## Results

Epidemiological and demographic characteristics of children included in the analysis are detailed in Table 2. Among these 1,859 children, about half resided in Brazil's Southeast region. The majority was aged 1-5 years at diagnosis, with more than two thirds still alive at the end of follow-up. In total, 1,321 (75.2%) of cases were diagnosed at an advanced staged as defined by category "C". Most cases had their AIDS diagnosis established between 1996-2002 and at some point used ART for HIV infection treatment during follow-up, mainly during 1996-2002. A high proportion of mothers had received prenatal care and breast-fed; MTCT preventive measures were not used in the majority of cases (Table 2).

**Table 2:** Epidemiological and demographic characteristics of children, and bivariate analysis of factors associated with first event of opportunistic illnesses

		Total N (%)	Opportunistic illness N (%)	OR (95% CI)	p
Total	-	1859 (100.0)	1218 (65.5%)	-	-
Study cohort	1983-1998	914 (49.2)	738 (80.7)	ref	
	1999-2002	945 (50.8)	480 (50.8)	0.25 (0.20-0.30)	< 0.001
Brazilian region (residence)	North	107 (5.8)	23 (21.5)	ref	
	Northeast	288 (15.5)	122 (42.4)	2.68 (1.62-4.46)	< 0.001
	Southeast	931 (50.1)	704 (75.6)	11.33 (7.51-17.09)	< 0.001
	South	395 (21.2)	267 (67.6)	7.62 (4.78-12.13)	< 0.001
	Central-West	138 (87.4)	102 (73.9)	10.35 (5.89-18.18)	< 0.001
Gender	Male	909 (48.9)	594 (65.3)	ref	
	Female	950 (51.1)	624 (65.7)	1.02 (0.84-1.23)	0.88
Birth cohort	< 1988	155 (8.3)	127 (81.9)	ref	
	1989-1995	819 (44.1)	582 (71.1)	0.54 (0.35-0.83)	0.006
	1996-2002	885 (47.6)	509 (57.5)	0.30 (0.20-0.45)	< 0.001
Age Group, HIV diagnosis	< 1 year	667 (35.9)	515 (77.2)	2.56 (1.81-3.61)	< 0.001
	1-5 years	1020 (54.9)	605 (59.3)	1.10 (0.79-1.53)	0.62
	6-12 years	172 (9.3)	98 (57.0)	ref	
Year, HIV diagnosis	< 1988	35 (1.9)	28 (80.0)	ref	
	1989-1995	544 (29.3)	449 (82.5)	1.18 (0.50-2.78)	0.65
	1996-2002	1280 (68.9)	741 (57.9)	0.34 (0.15-0.76)	0.009
ART use	Yes	1555 (83.6)	990 (63.7)	0.58 (0.44-0.77)	< 0.001
	No	304 (16.4)	228 (75.0)	ref	
Year, ART initiation	< 1996	314 (20.2)	259 (82.5)	ref	
	1996-2002	1241 (79.8)	731 (58.9)	0.30 (0.23-0.41)	< 0.001
Children status	Alive	1226 (68.9)	695 (56.7)	0.26 (0.20-0.33)	< 0.001
	Dead	554 (31.1)	463 (83.6)	ref	
Prenatal care	Yes	818 (76.6)	533 (65.2)	1.07 (0.80-1.44)	0.65
	No	250 (23.4)	159 (63.6)	ref	
Breast-feeding	Yes	969 (78.7)	628 (64.8)	0.92 (0.69-1.22)	0.56
	No	262 (21.3)	175 (66.8)	ref	
MTCT prophylaxis	Yes	254 (15.3)	139 (54.7)	ref	
	No	1404 (84.7)	922 (65.7)	1.58 (1.21-2.07)	0.001

About two thirds of children presented opportunistic infections during the observation period with a significantly lower occurrence of at least one of these events in the second cohort (OR 0.25, 95% CI 0.20-0.30 p < 0.001). The bivariate analysis of factors associated with the occurrence of opportunistic infections is depicted in Table 2. The most important factors associated were: region of residence within in Brazil (OR 2.68-11.33, as compared to the Northern region), low age at diagnosis (OR 2.56), and performance of MTCT prevention measures (OR 1.58). The use of ART has a protective effect. Children of the second cohort study and those diagnosed after HAART availability also developed significantly less opportunistic illnesses. Gender, pre-natal care and breastfeeding were not significantly associated with the occurrence of opportunistic diseases (Table 2).

Among the 1,218 children with at least one opportunistic disease, in most cases this disease was defined by presumptive diagnosis: 651 children (88.2%) in the first cohort, and 383 (79.8%) in the second (OR 1.90, 1.39-2.59, p < 0.001). The occurrence of specific opportunistic illnesses is presented in Table 3. In both pre-HAART and HAART eras, bacterial infections (excluding meningitis and sepsis) were the most frequent events (58.6% vs. 34.7%), followed by *P. jirovecii *pneumonia (21.9% vs. 13.2%) and bacterial meningitis/sepsis (16.8% vs. 7.4%). We observed a decreased proportion of cases in the second cohort, as compared to the period < 1988. Considering the pre-HAART and HAART eras, a significant decrease was also observed (OR 0.29, 0.23-0.37, p < 0.001) (Table 3).

**Table 3:** Occurrence of first event of opportunistic illnesses in the two Brazilian national cohorts of children with AIDS (n = 1,859)

		Cohort-HIV Diagnosis			
				
Opportunistic illness	Total N (%)	Pre-HAART Era N (%)	HAART Era N (%)	OR (95% CI)	P
Invasive bacterial infection (excluding meningitis and septicemia)	791 (42.6)	359 (58.6)	432 (34.7)	2.66 (2.19-3.24)	< 0.001
*P. jirovecii *pneumonia	298 (16.0)	134 (21.9)	164 (13.2)	1.85 (1.44-2.37)	< 0.001
Bacterial meningitis or sepsis	195 (10.5)	103 (16.8)	92 (7.4)	2.53 (1.89-3.39)	< 0.001
Wasting syndrome	179 (9.6)	91 (10.0)	88 (9.3)	1.09 (0.80-1.48)	0.637
HIV encephalopathy	145 (7.8)	54 (8.8)	91 (7.3)	1.23 (0.86-1.74)	0.270
Cytomegalovirus infection	106 (5.7)	40 (6.5)	66 (5.3)	1.25 (0.83-1.87)	0.289
Candidiasis	101 (5.4)	35 (5.7)	66 (5.3)	1.08 (0.71-1.65)	0.744
Cryptosporidiosis	50 (2.7)	36 (5.9)	14 (1.1)	5.49 (3.13-9.62)	< 0.001
Herpes simplex, gingivostomatitis	39 (2.1)	20 (2.2)	19 (2.0)	1.09 (0.58-2.06)	0.872
Cerebral toxoplasmosis	33 (1.8)	13 (2.1)	20 (1.6)	1.33 (0.66-2.68)	0.457
Herpes simplex, mucocutaneous	25 (1.3)	10 (1.6)	15 (1.2)	1.36 (0.61-3.04)	0.521
Disseminated mycobacterial disease	22 (1.2)	9 (1.5)	13 (1.0)	1.41 (0.60-3.31)	0.494
Isosporiasis	21 (1.1)	11 (1.2)	10 (1.1)	1.14 (0.48-2.69)	0.829
Lymphoma	17 (0.9)	7 (1.1)	10 (0.8)	1.43 (0.54-3.75)	0.450
Cryptococcosis	15 (0.8)	10 (1.6)	5 (0.4)	4.12 (1.52-11.14)	0.010
Kaposi's sarcoma	3 (0.2)	2 (0.3)	1 (0.1)	4.08 (0.44-37.42)	0.254
Leukoencephalopathy	3 (0.2)	3 (0.5)	0 (0.0)	-	-
Salmonellosis	2 (0.1)	1 (0.2)	1 (0.1)	2.03 (0.13-30.79)	0.551
Histoplasmosis	0 (0.0)	0 (0.0)	0 (0.0)	-	-
Coccidioidomycosis	0 (0.0)	0 (0.0)	0 (0.0)	-	-

The analysis related to HIV infection diagnosis in the pre-HAART and HAART eras shows a statistically significant reduction for invasive bacterial infection meningitis, *P. jirovecii *pneumonia, bacterial meningitis or sepsis, cryptosporidiosis, and cryptococcosis (Table 2). There were no reported cases of disseminated coccidioidomycosis and disseminated histoplasmosis in both periods, and no cases of progressive multifocal leukoencephalopathy in the HAART era (Table 3).

## Discussion

This study represents the first population-based evidence regarding the occurrence and trends of opportunistic illnesses in Brazilian children with AIDS [12,16]. The second national cohort study showed a higher proportion of children exposed to HAART, as compared to the first study (65% vs. 35%). However, despite the significant reduction, opportunistic illnesses still were important for AIDS-related morbidity, with more than half of the children with at least one event in the more recent cohort.

The impact of HAART on the reduction of progression to AIDS in children has been described repeatedly [2,3,5,8,10,22-24], even in developing countries [25-27]. However, few population-based studies have evaluated changes in the pattern of occurrence of diseases associated with HIV/AIDS [11,18,28,29]. Our data show a significant reduction in occurrence of all opportunistic illness in the HAART era: children that were born, or diagnosed with HIV/AIDS or had ART initiation after 1996 consistently and significantly reducing the chances of having opportunistic illness. However we identified a persisting relative importance of some specific events such as invasive bacterial diseases. A study conducted in Italy in a cohort of 1,402 children exposed to HIV through MTCT and representative of the pre-HAART (1985-1995) and HAART (1996-2000 and 2001-2005) periods showed the progressive reduction in the rate of specific clinical events, similar to our study with persisting high rates of serious bacterial infections, particularly pneumonia [11]. In Brazil, a previous study conducted in Minas Gerais State (Southeast region) in a referral center showed that the effectiveness of HAART was associated with significant reduction in the incidence of opportunistic infections in 371 children observed between 1989 and 2003 [18]. However, these data were not representative for the AIDS population.

In contrast to these previous studies, we observed that about 1/7 of children still presented *P. jirovecii *pneumonia in the HAART era reflecting the late diagnosis and management of HIV infection, despite the significant reduction in the incidence of this event. In our study cryptosporidiosis was significantly reduced (more than five times), as observed in other studies [11,29]. For other diseases (excluding cryptococcosis), despite the indication of reduction in the proportion of cases in the second period, there was not a significant difference between the periods.

Children with AIDS diagnosed between 1996-2002 had a three times lower odds of having opportunistic infections, as compared to those born before the release of any ARV. Thus, they had the chance to be treated with ARV, even in advanced stages of HIV infection [6,13,14].

Operational issues related to health services examined in this study, as the realization of prenatal care and adoption of breastfeeding were not significantly associated with the occurrence of opportunistic events, the first guidelines for management of HIV infection and opportunistic diseases were published in 1994, and the first therapeutic consensus was established in 1996. Expanded further actions for the prevention of MTCT have occurred since 1999 [6]. Despite these advances in national policy for control of HIV [13,30], there are indications of operational failures in these procedures [30-32]. Additionally, regional differences are important factors that contribute in Brazil to sustain a non-favorable outcome [16,17,30]. Our study points to a statistically significant association between non-performance of any measure aimed at the prevention of MTCT (prenatal or childbirth or child) and the occurrence of these clinical events. Approximately one seventh of the children had undergone some of the interventions to prevent MTCT, but still acquired HIV and developed AIDS. Probably, ongoing care and routine monitoring of children exposed to HIV would have prevented opportunistic illnesses in these cases. The vast majority of children in this study was born after 1995 (and about half of them after 1997), after wide implementation of MTCT control measures, evidencing a missed opportunity to prevent HIV transmission and to start HAART early.

We identified a positive association between the occurrences of opportunistic illnesses in all regions of the country compared with the North region, mainly in the Southeast where the epidemic is consolidated since the very beginning [30]. It is noteworthy that the North region had the lowest proportion of AIDS cases in the two periods (2% vs. 9%). After the period analyzed in these two cohorts, the North had a progressive increasing of incidence and mortality rates related to AIDS, which may have led to changes in the relative occurrence of these events when comparing regions [13,33].

Children aged < 1 year of age had a higher frequency of opportunistic illness compared with children aged 1-5 and 6-12 years. This population of children is known to progress more rapidly to AIDS, with strong evidence of efficacy of early treatment [4,8,34], which indicates the need for earlier interventions for the treatment of HIV infection. Based on this evidence, since 2009 Brazil has adopted measures on their therapeutic guidelines recommending the treatment of all HIV infected children < 1 year of age regardless of clinical symptoms, immunological classification or HIV viral load [35].

This study is based on the two unique national cohorts of children with AIDS for survival analysis with a representative number of children with AIDS in the country and its regions. Considering the sheer magnitude of the analyzed period (1983-2007), measures adopted for data collection were standardized, with a systematic review of all data collected, and minimizing the effects related to changes in criteria for AIDS cases definition, management/diagnosis of opportunistic infections and ART.

The present study has limitations beyond those already identified and described extensively in our previous publications on the topic [14-17]. Children in both cohorts had differing criteria for AIDS case definition, with greater sensitivity in the last one [6]. This may underestimate the occurrence of opportunistic diseases in this cohort. The presumptive diagnosis in health services may be related to difficulties of clinical diagnosis in some cases, the nature of each specific disease, technical conditions, such as provision of technical conditions for complementary diagnosis and experience of medical professionals with HIV/AIDS in children. Moreover, issues such as underreporting of clinical events and drop-out of some children during the periods specified in the two survival studies may have biased our results.

Considering the study period (1983-2007) and the advance of the Brazilian policy for the control and management of HIV infection/AIDS even after the introduction of HAART, the period after 2002 may have possibly a different pattern of occurrence of opportunistic conditions, indicating the need for further studies.

## Conclusions

The present study provides evidence for the reduction of morbidity related to AIDS-defining opportunistic illnesses in Brazilian children exposed to HIV through MTCT. This result reflects a period of further consolidation of HAART in a developing country with extensive commitment of the public sector to providing treatment. Bacterial diseases remain the most important opportunistic disease. Our study emphasizes the need to continually expand efforts to prevent MTCT of HIV, early diagnosis of infection, and improved care of pediatric AIDS patients.

## Competing interests

The authors declare that they have no competing interests.

## Authors' contributions

ANRJ participated in the study concept and design, had full access to all of the data, carried out acquisition, analysis, and interpretation of the data, drafted the manuscript. LHM participated in the study conception and design, and acquisition of data; had full access to all of the data, carried interpretation of the data, revising critically the manuscript. NH carried out interpretation of the data, revising critically the manuscript. JH participated in the study concept and design, had full access to all of the data, carried out analysis, and interpretation of the data, drafted the manuscript. All authors read and approved the final manuscript.
